# Wuzi Yanzong Pill for the treatment of male infertility: A protocol for systematic review and meta-analysis of randomized controlled trials

**DOI:** 10.1097/MD.0000000000021769

**Published:** 2020-08-14

**Authors:** Shanshan Yong, Yali Yang, Fuhao Li, Hangyu Yao, Fang Yang, Degui Chang

**Affiliations:** a Hospital of Chengdu University of Traditional Chinese Medicine; b Department of Andrology, The Reproductive and Women-Children Hospital, Chengdu University of Traditional Chinese Medicine, Chengdu, Sichuan Province, China.

**Keywords:** male infertility, protocol, systematic review, traditional Chinese medicine, Wuzi Yanzong Pill

## Abstract

**Background:**

The incidence of male infertility is increasing worldwide, and has become an important problem that plagues many married couples. Half of the infertility cases have induced by male infertility. Wuzi Yanzong Pill is a traditional Chinese herbal formula used in treating spermatorrhea, premature ejaculation, erectile dysfunction, lumbago and male sterility widely. Therefore, in this systematic review, we design to evaluate the effectiveness and safety of Wuzi-Yanzong Pill for the treatment of male infertility.

**Methods:**

The English and Chinese literature published before June 30, 2020 will be searched in PubMed, EMBASE, Cochrane library, and Chinese literature in China National Knowledge Infrastructure, Chinese biomedical document service system, VIP Chinese Science and Technology Journal Database, WANFANG data. All related randomized controlled trials that meet the eligibility criteria will be included and other studies will be excluded. We will search literature with text keywords “male infertility” or “sperm” or “semen” and “Wuzi Yanzong Pill” or “Wuziyangzong” or “WZYZ”. Progressive motility, sperm concentration, sperm morphology, sperm viability, sperm DNA fragmentation, sperm number per ejaculate, pregnancy rates will be evaluated. RevMan 5.3 and Stata 14.0 will be used to conduct this systematic review. The preferred reporting items for systematic reviews and meta-analysis protocols statement is followed in this protocol and the PRISMA statement will be followed in the completed systematic review.

**Conclusion:**

The efficacy and safety of Wuzi Yanzong Pill in the treatment of male infertility will be e evaluated. The results of this review may provide some help for the clinician's decision.

## 1 Introduction

Infertility is a worldwide problem and defined as the failure to achieve spontaneous pregnancy after 1 year of regular intercourse without any contraception.^[1]^ About 15% of couples are impacted by infertility and nearly half of them account for male's factors in general.^[2]^ There are many elements for male infertility patients, less than 10% with congenital or genetic abnormalities and 20% to 30% with pathogenic statuses such as anti-sperm antigen, varicocele, or infection, 30% to 50% male cannot find the pathogeny of infertility (idiopathic infertility). Currently, male infertility treatment methods are various, including antioxidants and assisted reproductive technologies such as IUI, IVF, and ICSI.^[3–5]^ Nevertheless, there is a lack of effective and specific pharmaceutical treatments for male infertility.

Based on traditional Chinese medicine (TCM) theory, infertility be classified as the category of “no child”, “sterility”. TCM believes that the kidney stores essence which controls development and reproduction in human life. Physicians of different dynasties believe that the prosperity and decline of kidney essence determine male's fertility, kidney insufficiency is the main pathogenesis of semen abnormality.^[6]^ Therefore, the therapeutic principle should be to focus on tonifying kidney and nourishing essence. In China, traditional herbal prescriptions, the basic form of clinical application of TCM for thousands of years, have been proven by clinical practice to play a positive role in human fertility. Wuzi Yanzong (WZYZ) Pill is the most common Chinese herbal formulas prescription for the treatment of male infertility. This prescription is known as the “The first prescription of ancient and modern infertility treatment ”, was first documented in the “ She Sheng Zhong Miao Fang ” edited by Shi-che Zhang in the Ming Dynasty. WZYZ pill is consists of Fructus Lycii, Semen Cuscutae, Fructus Rubi, Schisandra Chinensis, and Semen Plantaginis.

There are relatively many clinical reports that have found that WZYZ pill has a significant therapeutic effect on infertility. For instance, it is confirmed that WZYZ pill can significantly elevate the semen volume and sperm density in infertility patients with low semen counts.^[7,8]^ However, its quality and efficacy have not been systematically evaluated, which affects the reliability of the research conclusion. This brings confusion to the clinical application for clinicians. Therefore, it is necessary to carry out a systematic review and meta-analysis to fully evaluate the efficacy and safety of WZYZ Pill in the treatment of male infertility.

## 2 Review objectives

The purpose of this systematic review is to evaluate the efficacy and safety of WZYZ Pill for the treatment of male infertility, including sperm progressive motility, sperm concentration, sperm morphology, sperm activity rate, sperm DNA fragmentation, sperm number per ejaculate, pregnancy rates, provide evidence-based medical evidence and suggestion for andrologists and urologists in the future.

## 3 Methods

This is a systematic review, and the meta-analysis will be carried out as conditions permit. Since this is a systematic review based on original research, no ethics committee approval is required.

### 3.1 Protocol and registration

This protocol has been registered on the International Platform of Registered Systematic Review and Meta-analysis Protocols (registration number: INPLASY202070046) which could be available on https://inplasy.com/. The preferred reporting entries of the PRISMA statement for system review and meta-analysis protocols (PRISMA-P) will be followed in this protocol.^[9,10]^ And the PRISMA statement will be followed when reporting the systematic review.

### 3.2 Eligibility criteria

The inclusion and exclusion criteria are summarized as follows.

#### 3.2.1 Study designs

The study will include only randomized controlled trials (RCTs). All the case reports, patient series, retrospective studies, self-controlled or before and after controlled studies, animal studies, reviews, laboratory researches, observational studies, meta-analyses, letters and other secondhand studies will be excluded.

#### 3.2.2 Participants

##### 3.2.2.1 Included population

The infertile patients must be older than 18 years old, who were at least 1 year of unprotected sexual intercourse without contraception, and healthy female partners (their tubal, uterine, cervical abnormalities, and ovarian disorders were excluded). The patients should be conforming to the diagnostic criteria established in the European Urological Association's 2012 edition^[2]^ or other authoritative standards.

##### 3.2.2.2 Excluded population

Healthy people; undiagnosed patients; female infertility patients; azoospermia; infertility reason for obstructive diseases, hypothalamic-pituitary lesion, chromosomal or genetic lesion, endogenous or exogenous hormone abnormalities, congenital abnormality.

#### 3.2.3 Interventions

This group was treated with WZYZ pill or it combines with Western medicine are used as treatment interventions, limited to RCTs for drug therapy. If WZYZ pill is used as a control in the trial and another drug is an intervention, we consider reversing the order of the 2 interventions in this systematic review, which means WZYZ pill will be regarded as an intervention measure and the other drug as a control measure.

#### 3.2.4 Control interventions

The control interventions can be accepted simple western medicine or didn’t get any treatment as a blank control. But, once they had accepted other traditional Chinese medicine treatments such as intravenous medication, acupuncture, and moxibustion, the trials will be excluded.

#### 3.2.5 Outcomes

##### 3.2.5.1 Primary outcome indicator

(1) Progressive motility sperm: including the activity of A and B levels or forward-moving sperm in the World Health Organization classification, which provided as a percentage (%).
(2) Sperm concentration: number of sperm per milliliter (10^6^/mL).^[4]^
(3) Sperm morphology: proper sperm ratio, provided as a percentage (%).
(4) Sperm viability: Proportion of all active sperm (including A, B, C or PR, NP), provided as a percentage (%).

It will be based on the results reported at the end of included studies.

##### 3.2.5.2 Secondary outcome indicators

(1) Sperm DNA fragmentation: DNA integrity damage was reported in the study. The detection method may be sperm chromatin structure assay (SCSA), terminal deoxyuridine nick end labelling (TUNEL) assay, Comet assay, sperm Chromatin Dispersion (SCD) assay, Acridine orange (AO) test, Aniline blue (AB) staining, Toluidine blue, Chromomycin A3 (CMA3) staining.^[11]^
(2) Sperm number per ejaculate: The total number of sperm contained in once ejaculation (10^6^/once ejaculation).
(3) Pregnancy rate: defined as all pregnancy reported in the study.
(4) Adverse events: all adverse events, including nausea, vomiting, facial flushing, increased heart rate and other adverse events in the study.

### 3.3 Data source

#### 3.3.1 Electronic search

We will search in PubMed, EMBASE, Cochrane library, and Chinese literature in China National Knowledge Infrastructure (CNKI), Chinese biomedical document service system (Sino Med), VIP Chinese Science and Technology Journal Database (VIP), WANFANG data. The literature publication deadline is June 30, 2020, in each platform or database and the search work will be done in July 2020. The literature search update will be executed again before the systematic review is completed.

Subject heading, free text words will be used to search in PubMed, EMBASE, Cochrane library. In Cochrane Library and EMBASE, the use of free words will be limited within title, abstract and keywords, but in PubMed, limited in title/abstract. The “topic” field will be used for the search of CNKI and WANFANG, and the “title or keyword” filed for the search of VIP. The subject heading plus free words form will be used to retrieve SinoMed.

We will choose Medical Subject Heading or text key words “male infertility” or “sperm” or “semen” AND “Wuzi Yanzong” or “Wuzi Yangzong Pill” or “WZYZ”. The Chinese form of the above terms will be used in Chinese search. A specific search example for PubMed is shown in Table 1.

**Table 1:**
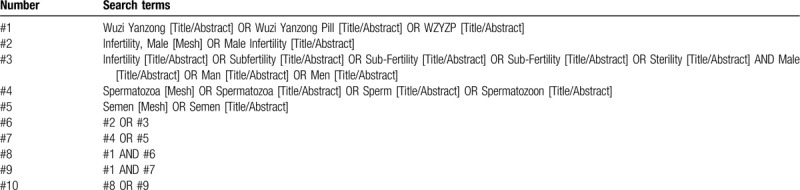
PubMed search strategy.

#### 3.3.2 Other sources of search

Grey literature will be retrieved through Open Grey. Full texts will be obtained through library interlibrary loan or purchase. The manual review of references in published articles will be conducted to identify other relevant studies.

### 3.4 Selection of studies and data extraction

#### 3.4.1 Selection of studies

Document management will be conducted by Endnote X8 software. The software will be used to filter duplicate studies first, and then delete duplicate researches by reading titles, abstracts and other relevant information.

According to the Included and excluded population, the literature will be further screened. In this process, the controversial literature will be screened after obtaining the full text. Further detailed screening and data extraction of the documents will be carried out simultaneously by 2 professionally trained reviewers (Shanshan Yong, Yali Yang).

**Figure 1:**
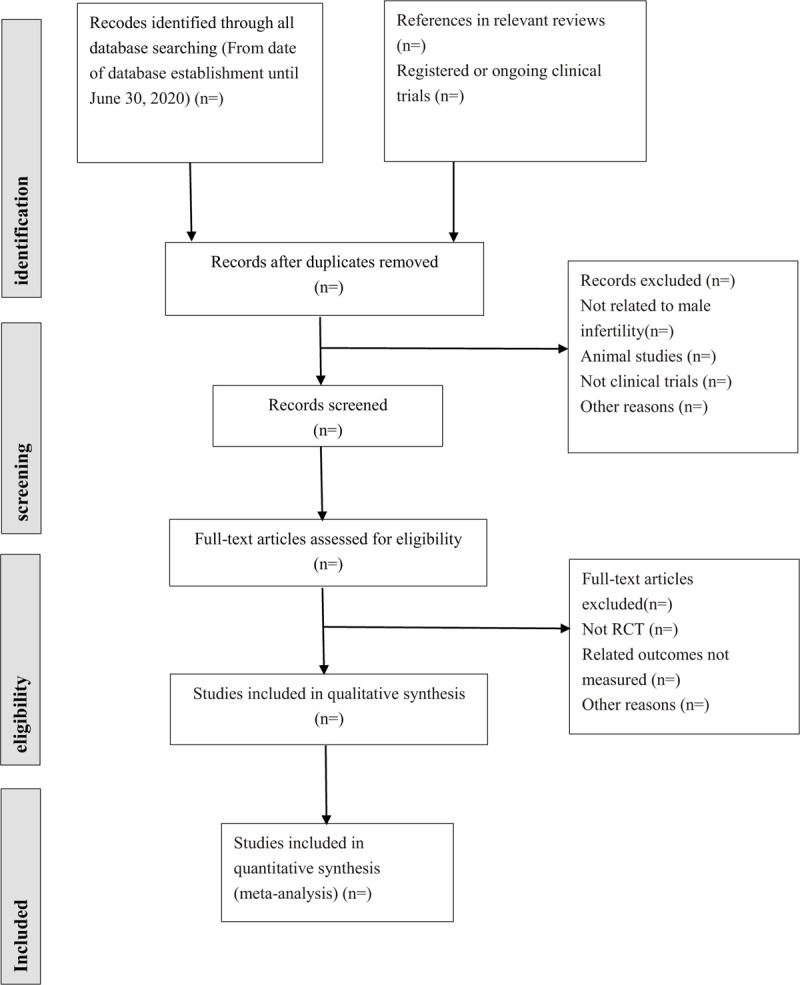
The preferred reporting items for systematic review and meta-analysis protocols (PRISMA) literature screening flow chart.

Then, the articles that meet the inclusion criteria are full-text read and re-screened. If 2 or more articles have repeated or staged research results, only the articles with the largest sample size, the most complete intervention and follow-up time are included. When the review team cannot confirm the repeated studies, the original study author will be contacted for judgment. The flow chart of literature screening is shown in Figure 1.

#### 3.4.2 Data extraction

Before the formal process of data extraction, the review group will discuss and a unified data extraction form will be produced. Two review authors (Shanshan Yong, Yali Yang) will independently conduct data extraction exercises. All differences will be discussed and resolved with the third reviewer (Fuhao Li). The content of data extraction is as follows.

1. General characteristics: name of first author, publishing year, study title, nation or country, execution time of the study, e-mail or other contact information.
2. Information of studies: study design, sample size, randomized information, assignment hiding, blind method, diagnostic criteria, outcome indicators, safety indicators, statistical methods, information of outcome indicators, follow-up.
3. Information of participants: age, severity of disease, course of disease, baseline level, comorbidity, healthy condition.
4. Information of control group: The packaging, shape, taste and color of the oral drug should be consistent with that of the treatment group, and neither the researcher nor the participants could distinguish them.
5. Outcome indicators: Detailed statistics of sperm quality parameters, including sperm viability, progressive motility, sperm concentration, sperm morphology, sperm DNA fragmentation, Sperm number per ejaculate and pregnancy rates, data of adverse events, specific information.
6. Risk of bias: random sequence generation, allocation concealment, blinding of participants and personnel, incomplete outcome data, selective reporting, other bias.
7. Other study information: funding situation, conflict of interest.

When necessary, the review team will contact the original research author by email to obtain the full text or relevant results. If there are any questions or confusion about the original research in the process, we will be contacted again to get specific answers.

### 3.5 Risk of bias assessment

There are 2 review authors (Shanshan Yong, Yali Yang) who will independently evaluate and cross-check the risk of bias: including selection bias, performance bias, detection bias, attrition bias and reporting bias, which will be evaluated based on the Cochrane Collaboration Network Risk Assessment Tool. Discrepancies between review authors on the risk of bias will be resolved through discussion with a third review author (Fuhao Li). Assessment items include random sequence generation, allocation concealment, blinding of participants and personnel, blinding of outcome assessment, incomplete outcome data, selective reporting and other bias. Each item of bias situation includes low risk, unclear and high risk.^[12]^ Since the authenticity of blindness cannot be determined, the outcome indicators of the systematic review are relatively objective. Therefore, we define the generation of random sequence, allocation concealment and incomplete data as the key areas of bias assessment risk. The risk of bias assessment chart containing the study will be generated using the Review Manager 5.3 software.^[12]^

### 3.6 Data analysis and synthesis

Descriptive analysis or narrative synthesis will be performed. When there is clinical heterogeneity between the studies or when the data cannot be synthesized or results data cannot be extracted. When the included trials are clinically homogeneous and the data are similar and synthesizable, a meta-analysis will be performed.^[13,14]^ Dichotomous will be determined by relative risk (RR) with 95% confidence interval (CI).^[13]^ Continuous data will be analyzed using weighted mean difference (if measurement methods are consistent) or standardized mean difference (if measurement methods are different). We will use Cochran's *Q* statistic and *I*^2^ statistic to test heterogeneity. *P* < .10 is heterogeneous, or *I*^2^ > 50% is significant heterogeneity. A fixed effects model (Mantel-Haenzel method for RR and Inverse Variance for MD) will be used for *I*^2^ < 50%. A random effects model (d–l method)^[13]^ will be used when the heterogeneity is still significant after sensitivity analysis and subgroup analysis. *P* < .05 of Z test will be considered statistically significant. The meta-analysis will be generated by Review Manager 5.3 software.^[13,14]^

### 3.7 Subgroup analysis

If the data is sufficient and there is heterogeneity between studies, we will perform subgroup analysis. Subgroup analysis will be conducted according to different ages, ethnic groups, male infertility types, comorbidity, interventions, control measures, measurement methods or measurement time.

### 3.8 Sensitivity analysis

Sensitivity analysis will be used to test the stability and reliability of meta-analysis. It can be done by eliminating each study individually or using random-effects model (D-L method) to test the results after using the fixed effect model.

### 3.9 Publication bias

We will use a funnel plot to test the risk of publication bias if a result of a meta-analysis contains >10 articles and above. Quantitative methods such as Begg test and Egger test will be used to help assess publication bias in the application.

### 3.10 Grading the quality of evidence

The quality of evidence in the systematic review will be judged by the GRADE tool.^[15]^ According to 5 key domains: risk of bias, consistency, directness, accuracy and publication bias. The level of evidence for each outcome can be divided into high quality, moderate quality, low quality and very low quality levels.^[16]^

## 4 Discussion

In recent years, infertility has received increasing attention. Due to environmental pollution, or long-term lack of trace elements, unhealthy living habits, long-term mental stress, excessive smoking, drug abuse, abuse of hormone drugs, sexually transmitted diseases and other factors, the reproductive capacity of humans, has shown a significant downward trend, particularly in men.^[17]^ Currently, there are diverse drugs recommended to treat male infertility, but medication is mainly experiential therapy.

Traditional Chinese medicine pays attention to the theory of the concept of holism and syndrome differentiation, which is to be considered as a safe and effective method in the treatment of infertility. The pathological mechanism of male infertility in TCM is asthenia, sthenia, cold, heat, phlegm, blood stasis and depression, which eventually leads to kidney essence deficiency.^[18,19]^

WZYZ pill is composed of 5 traditional Chinese herbs, exert the effect of nourishing kidney essence jointly. WZYZ pill primarily acting on testicular spermatogenic epithelial cells, which directly affects the differentiation and development of spermatogenic cells and restores spermatogenic function.^[20]^ It has been shown that WZYZ pill can significantly improve the semen quality and increase serum testosterone (T) and luteinizing hormone (LH) levels in male infertility patients.^[7,21]^ WZYZ pill can remarkably enhance male semen parameters. The results show that WZYZ pill combined with levocarnitine for treatment of oligoasthenospermia could elevated sperm quality and sperm motility.^[22]^ The study has shown that WZYZ pill can improve semen parameter indexes of patients with sperm DNA damage, reduce the rate of sperm DNA fragmentation, and have a repairing effect on sperm DNA damage.^[23]^ WZYZ pill is a traditional Chinese formula that has been making use of the treatment of male infertility for a long time. There are many relatively clinical reports testified its clinical effect is remarkable. However, the experimental quality and conclusion of these researches are not well substantiated, which affects the reliability of the studies, and it is difficult to be recognized in the clinical application of clinicians. Therefore, this meta-analysis aims to evaluate the therapeutic effect of WZYZ pill on male infertility patients through only select randomized controlled trials (RCT). We will assess the effect of WZYZ pill on semen parameters including Progressive motility, sperm concentration, sperm morphology, sperm viability and sperm DNA fragmentation, sperm number per ejaculate, pregnancy rates in infertile men. In conclusion, this systematic review will provide evidence-based medical evidence to prove the effectiveness and safety of WZYZ pill in improving the reproductive outcome and reproductive capacity of male infertility, and provide recommendations for further researches.

This systematic review also has some limitations: First, there may not be enough large samples of RCTs. Second, the quality of some RCTs may not be high, which will affect the authenticity of the evidence. Therefore, we will hope there will have more large-scale, rigorous, high-quality and reasonable multicenter randomized controlled trials (RCTs) to explore the clinical efficacy of the treatment of male infertility, provide a more objective and well-founded conclusion in the future.

## Author contributions

**Conceptualization:** Shanshan Yong, Yali Yang, Degui Chang

**Data curation:** Shanshan Yong, Yali Yang, Fuhao Li, Hangyu Yao

**Formal analysis:** Shanshan Yong, Yali Yang, Fuhao Li

**Funding acquisition:** Shanshan Yong, Fang Yang

**Investigation:** Shanshan Yong, Yali Yang, Fuhao Li, Degui Chang

**Methodology:** Yali Yang, Fang Yang

**Project administration:** Shanshan Yong, Yali Yang, Fang Yang, Degui Chang

**Resources:** Fang Yang, Degui Chang

**Software:** Shanshan Yong, Yali Yang, Fuhao Li, Hangyu Yao

**Supervision:** Shanshan Yong, Yali Yang, Fuhao Li, Degui Chang

**Validation:** Shanshan Yong, Fang Yang, Degui Chang

**Writing – original draft:** Shanshan Yong, Yali Yang

**Writing – review & editing:** Fuhao Li, Hangyu Yao, Fang Yang, Degui Chang

DGC is the guarantor. All authors read, provided feedback and approved the final manuscript.
